# Characterizing zinc diffusion during liquid metal embrittlement of resistance spot welded TWIP steel

**DOI:** 10.1007/s40194-026-02384-4

**Published:** 2026-02-10

**Authors:** Gautham Mahadevan, Virginia Bertolo, Soheil Sabooni, Vera Popovich, Leo A. I. Kestens, Marcel Hermans

**Affiliations:** 1 Department of Materials Science and Engineering, Delft University of Technology, Mekelweg 2, Delft, 2628 CD The Netherlands; 2 Department of Electromechanics, Systems and Metals Engineering, Ghent University, Technologiepark 46, Ghent, 9052 Belgium; 3 Tata Steel Nederland Technology B.V., P.O. Box 1000, IJmuiden, 1970 CA The Netherlands

**Keywords:** Liquid metal embrittlement, LME, Resistance spot welding, Zn diffusion, Grain boundary diffusion, Bulk diffusion, Stress-assisted diffusion, TWIP steel, Finite element analysis

## Abstract

Liquid metal embrittlement (LME) during resistance spot welding (RSW) of twinning induced plasticity (TWIP) steel is primarily driven by stress-assisted grain boundary (GB) diffusion of zinc (Zn). Although GB diffusion is widely recognized as the dominant LME mechanism, experimental quantification is challenging due to resolution limitations. This study characterizes Zn diffusion in TWIP steel during RSW by conducting energy dispersive X-ray spectroscopy (EDS) line scans ahead of LME cracks in both the rolling direction (RD) and normal direction (ND) over weld times from 700 to 1700 ms. Results reveal that Zn diffusion distance increases with weld time, with consistently higher diffusion in the ND. To compare experimental measurements with diffusion theory, an FEA simulation based on Fick’s law was employed to approximate bulk Zn diffusion under varying temperatures. The model predicts Zn diffusion trends consistent with experimental observations. Although the diffusion distance predicted in the simulation exceeds measured values, directional trends are accurately captured. A theoretical framework to compare GB and bulk diffusion was proposed. GB diffusion distance of Zn is estimated to be approximately 30 times greater than bulk diffusion, establishing a quantitative link between weld time and Zn diffusion during RSW of TWIP steel.

## Introduction

Reducing fuel consumption and improving the carbon footprint in vehicle manufacturing has been an important objective for the automotive industry in recent decades. One effective approach to reducing fuel consumption is to decrease vehicle weight without compromising structural strength or crash resistance [1]. Advanced high-strength steels (AHSS) are well-suited for this purpose, offering the high strength and elongation properties needed for automotive applications. These materials enable the construction of stronger, crash-resistant and lighter vehicles, thus enhancing both safety and fuel efficiency [2].

Resistance spot welding (RSW) is the primary joining method used in the automotive manufacturing process, with each vehicle containing an average of 3000 to 5000 spot welds [3]. AHSS grades are typically coated with a Zn anti-corrosion layer. The combination of electrode force and Joule heating produces localized melting and solidification, leading to nugget formation. The RSW process can be controlled by setting welding parameters such as electrode force, welding current, squeeze time, weld time and hold time. Electrode geometry and tip radius are also critical factors controlling nugget growth and current density [4]. Each weld cycle in RSW consists of 3 stages. The first stage, known as the ‘squeeze time’, involves applying electrode force on the steel sheets, to ensure the sheets are firmly in contact before current is applied. This is followed by the ‘weld time’, during which the electrode force continues to be applied along with a weld current to the sheets to generate resistive heating that causes localized melting at the sheet-sheet interface. This forms a weld nugget at the interface, which is the region in the interface that melts during RSW. Finally, during the ‘hold time’, the current is stopped while the force is maintained by water-cooled electrodes, allowing a metallurgical bond to form between the sheets [5]. The temperature and stress conditions at the weld can cause the Zn coating to become liquid during welding and penetrate the GBs of the solid steel substrate. This penetration can result in the formation of cracks [6]. This is known as liquid metal embrittlement (LME) and poses a significant challenge for the use of AHSS such as twinning induced plasticity (TWIP) steel in automotive manufacturing, as each weld becomes a potential failure site [7]. Multiple studies have shown TWIP steel to have a high susceptibility for LME despite its superior strength and ductility [7–9]. The complexity of LME is increased by the inhomogeneous stresses, temperatures and microstructure across different regions of the spot weld. These variations lead to the formation of different types of LME cracks in different areas of the weld [10].

A number of theories have been proposed to explain the micro-mechanisms behind LME. These micro-mechanisms vary depending on the combination of metals in the LME system. For Fe-Zn, stress-assisted grain boundary (GB) diffusion has been observed to be the most likely micro-mechanism [11, 12]. The ‘Rehbinder effect’ underpins most of the proposed models for stress-assisted LME, which proposes that the liquid Zn penetrates the grain boundaries and reduces the surface energy of the steel which drives the embrittlement above a critical level of stress [13]. Multiple characterization studies have been performed on RSW showing LME in order to understand the effects of microstructure and temperature on crack initiation and propagation [6, 13–16]. These studies have quantitatively characterized Zn diffusion during LME. Kang et al. used STEM analysis to investigate Zn distribution along GBs ahead of LME crack tips and reported localized Zn penetration. While these measurements provide direct evidence of Zn penetration along GBs, they are limited to individual line scans at selected locations and cannot be extended across a wide range of welding parameters due to the complexity of sample preparation and the time-intensive nature of high-resolution characterization [17]. Similarly, Ling et al. attempted to quantitatively measure Zn content in LME-affected regions; however, limitations in characterization resolution prevented reliable quantification of Zn diffusion along GBs [13]. RSW process parameters also have an effect on LME, with studies showing that LME is more severe when the welding heat input is higher, resulting in more severe LME when the welding current or weld time is increased [13, 14, 18, 19]. While studying LME severity by analyzing the number of cracks and crack dimensions is useful, it offers an incomplete picture because it does not account for the underlying Zn diffusion mechanisms that drive crack formation and propagation [20]. Understanding the overall Zn diffusion, particularly its interaction with stress and temperature gradients, is crucial for a more comprehensive understanding of LME. Klinger et al. proposed a ‘diffusion wedge’ theory, which describes how the embrittling species diffuses along grain boundaries ahead of an LME crack, forming a wedge-like concentration profile. This theory provided a mathematical framework linking stress, temperature, GB and bulk diffusion [20]. Dohie et al. developed an empirical model on Zn diffusion in α-Fe based on experimental observations. While this model predicted the temperature-dependent diffusion constants of Zn in α-Fe, it did not account for the effect of stress on Zn diffusion [21]. DiGiovanni et al. investigated the influence of stress on Zn diffusion that formulated a modification of the Fisher equation for GB diffusion to account for the contribution of stress, that also incorporated the model developed by Klinger et al. [22, 23]. Although the variation in Zn diffusion with stress was investigated in the study by DiGiovanni et al., the influence of temperature was not experimentally validated.

The presence of liquid Zn at the crack tip and the solid state diffusion of Zn ahead of the crack tip are recognized as the most critical steps governing LME. However, there has been a lack of detailed characterization studies linking weld time to the diffusion of Zn ahead of the crack tip. GB diffusion, although dominant in LME mechanisms, is challenging to measure experimentally due to the limited resolution of techniques like energy dispersive X-ray spectroscopy (EDS), which cannot reliably capture Zn concentrations along narrow GB regions. Since GB diffusion and bulk diffusion are related through established models, such as Fisher’s model [22], analyzing bulk diffusion enables an indirect understanding of GB diffusion. Although previous studies systematically investigate the effect of welding process parameters on LME, the severity of embrittlement is only quantified by the number of cracks and crack depth and not on their effect on the microstructure or mechanism governing LME [13]. Moreover, most of the characterization studies induce LME on the steel samples using high-temperature thermo-mechanical testing [7, 17, 24, 25]. Although this technique is useful in studying LME, it does not accurately replicate the inhomogeneous temperature, stress or microstructural conditions during RSW, which strongly affects crack propagation [26]. The main goal of the research presented here is to systematically study the effect of welding time on the diffusion of Zn ahead of the crack tip during RSW. Understanding the effect of weld time on Zn diffusion would thus lead to a better understanding of LME mechanisms and the design of the welding process that could result in reducing LME susceptibility.

This study investigates the influence of RSW weld time on Zn diffusion by comparing Zn diffusion profiles ahead of the crack tip in the rolling direction (RD) and normal direction (ND) relative to the sample at different weld times. By examining diffusion behaviour in different directions and utilizing measurable bulk diffusion data, this study establishes a link between weld parameters, stress distribution and Zn diffusion in the context of LME mechanisms. Additionally, a simplified 2D analytical diffusion model is used to evaluate the comparative trends of Zn diffusion in the RD and ND directions and to provide a simple framework to compare experimentally measured bulk Zn diffusion distances under varying weld conditions. The purpose of this model is not to provide an exact description of the diffusion process but to validate the calculated Zn diffusion distances at different RSW weld times and enhance understanding of the directional dependence of diffusion behaviour. Although previous studies have investigated the effect of weld time on LME during RSW, this is the first quantitative study linking RSW weld time and Zn diffusion distance of TWIP steel. The novelty of this study is in the extensive range of weld times studied and the quantitative link established between GB and bulk Zn diffusion for Fe-Zn systems.

## Material and methods

### Materials

**Fig. 1:**
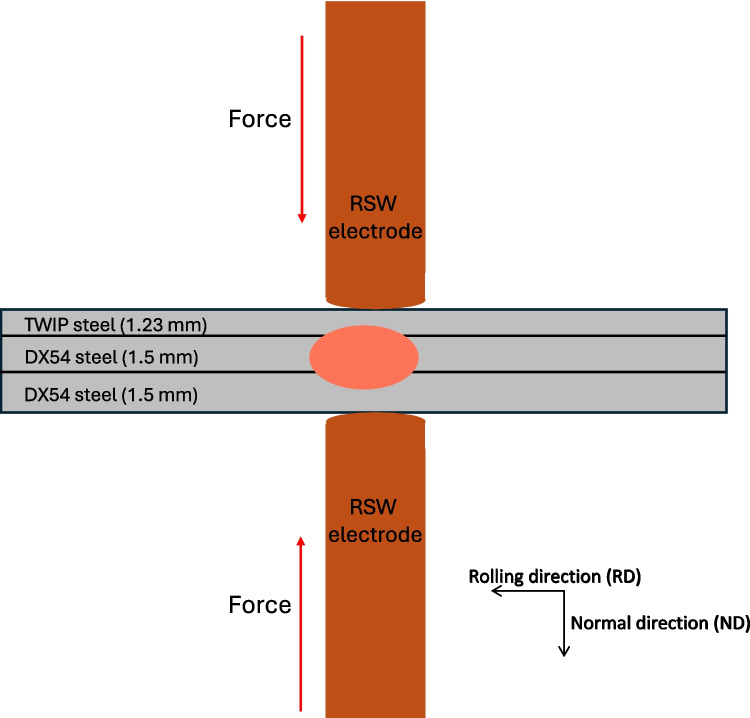
Schematic representation of the RSW setup showing the sample coordinate system

The investigated material was a sheet of LME-susceptible galvanized, cold-rolled austenitic TWIP steel with a thickness of 1.23 mm and a Zn coating thickness of approximately 12 µm. The TWIP steel was welded over two sheets of DX54 steel of thickness 1.5 mm and an average Zn thickness of approximately 15 µm to form a heterogeneous triple-stack weld, as shown in Fig. 1. DX54 steel was used as backing sheets in a triple-layer weld configuration to increase heat input and promote LME crack formation [15]. The composition of the main alloying elements in the TWIP steel is presented in Table 1.

**Table 1:** Composition of alloying elements in TWIP steel in wt%

Mn	Al	C	Ni
17.3	1.49	0.61	0.36

### Welding setup

Spot welds were produced on a 1000-MHz MFDC spot welding machine, using a constant current regulation. ISO5821 F1-16–20-6 CuCr1Zr electrodes were tip-dressed before use. The welding current, electrode force, squeeze time and hold time were kept constant, using the process parameters presented in Table 2, and were selected after internal optimization. The welding current was optimized to be the highest value that did not result in expulsion at a weld time of 1700 ms. Four samples were each made at 8 different weld times (WT) from 300 to 1700 ms at an interval of 200 ms.

**Table 2:** RSW process parameters

Welding current (kA)	Electrode force (kN)	Squeeze time (ms)	Hold time (ms)	Weld time (ms)
7.4	4.5	150	150	300, 500, 700, 900, 1100, 1300, 1500 and 1700

### Characterization

A VHX7000N optical microscope was used to identify LME cracks on the surface of the spot weld. These crack locations were used to select the areas to perform cross-section analysis. Once the cracks were identified, they were sectioned with the cutting plane passing through the middle of the crack. To prepare the samples for SEM analysis, the cross-sections were ground using progressively finer sandpaper and then polished sequentially with a 3-µm silica paste followed by a 1-µm diamond paste to achieve a smooth surface suitable for imaging and microscopy.

**Fig. 2:**
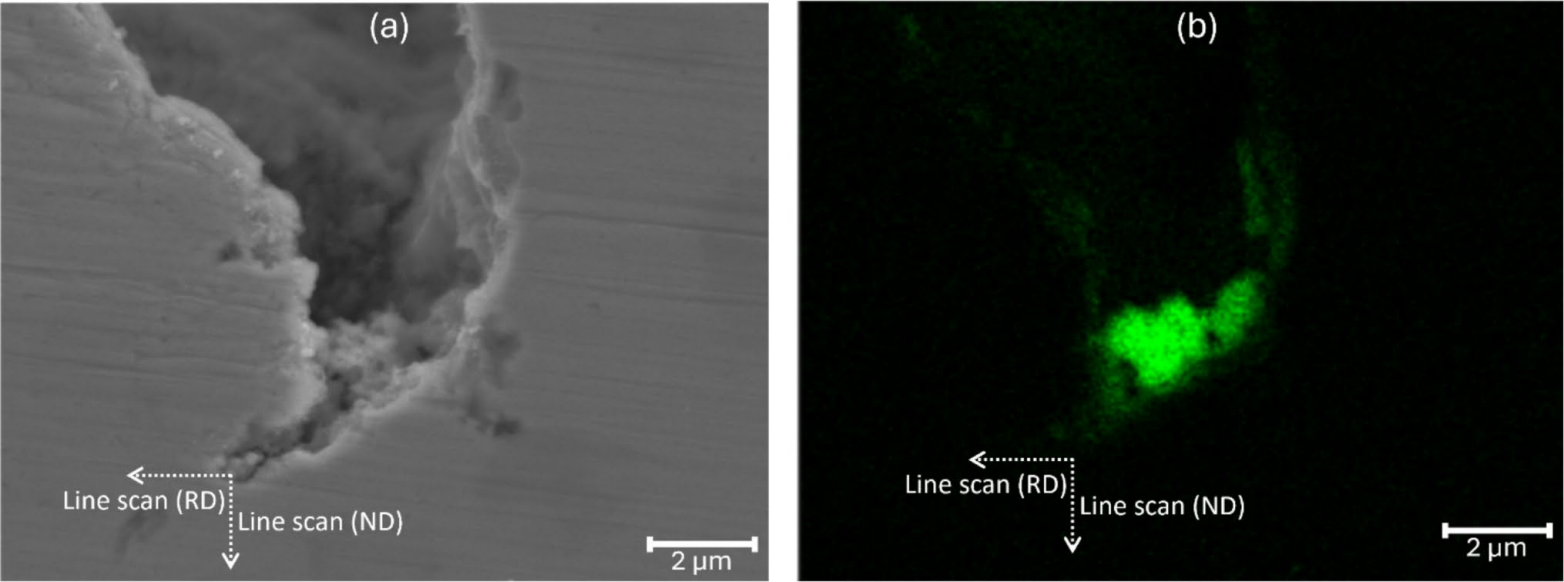
**a** SEM micrograph of a representative LME crack tip illustrating EDS line scan directions relative to the sample coordinate system. **b** EDS map showing distribution of Zn in the LME crack illustrating EDS line scan directions relative to the sample coordinate system

**Fig. 3:**
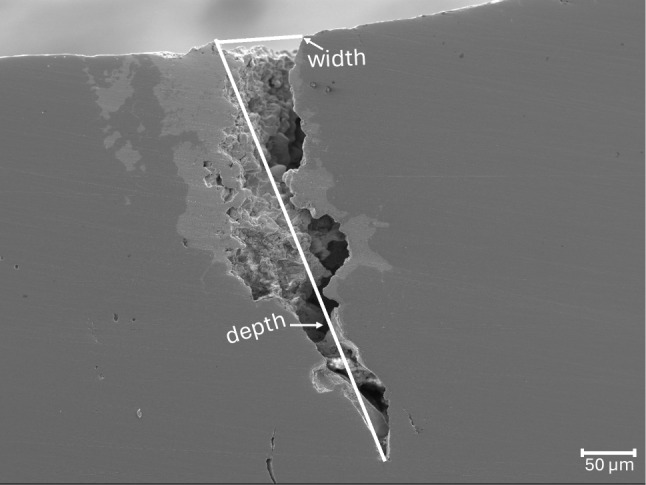
Schematic measurement of LME crack width and depth from the weld surface. The crack depth (indicated by the white line) is measured from the surface opening to the crack tip in the cross-section

Scanning electron microscopy (SEM) was conducted with a JEOL® IT800SHL™ equipped with a field emission gun at 10 kV and 3.2 nA beam current. The SEM system can perform energy dispersive X-ray spectroscopy (EDS) with an Oxford Instruments® Maxim 100™ detector. The primary objective of the EDS analysis was to investigate the bulk diffusion of Zn from the crack tip into the surrounding steel grains. This served two purposes: (a) to study the influence of weld time on Zn diffusion and LME cracking behaviour and (b) to validate the theoretical framework of stress-assisted diffusion. As illustrated in Fig. 2, EDS line scans were conducted in two principal directions relative to the sample reference system: the normal direction (ND) and the rolling direction (RD). All cracks were observed to propagate perpendicular to the weld surface along the ND. The line scans aimed to capture and compare the Zn diffusion profiles along these directions to identify any directional differences in the bulk diffusion behaviour. SEM was used to measure the width and depth of LME cracks. The width was measured at the crack opening and the depth was measured from the surface crack opening to the crack tip, as shown in Fig. 3.

### Numerical modelling framework

The purpose of this simulation is to approximate the bulk diffusion of Zn at different temperatures and compare the resulting diffusion profiles with those measured at LME cracks from RSW tests at varying weld times. Theoretical line scans of Zn concentration in both the ND and RD from the crack tip are calculated based on solving the 2D diffusion equation using a continuum finite element formulation of Fick’s second law [27] and compared with experimentally obtained EDS line scans of Zn bulk diffusion ahead of the crack tip. The model assumes that the LME crack remains fully filled with liquid Zn, acting as an infinite reservoir for diffusion, ensuring a constant Zn concentration at the crack interface. The crack dimensions are fixed throughout the simulation, focusing solely on bulk diffusion rather than dynamic crack propagation. The formation of Fe-Zn intermetallic phases is also not included in the model, allowing for a direct evaluation of Zn diffusion in the steel matrix. This simplification was necessary to isolate and compare bulk diffusion trends without the added complexity of dynamic phase transformations or material loss.

#### Initial and boundary conditions

**Fig. 4:**
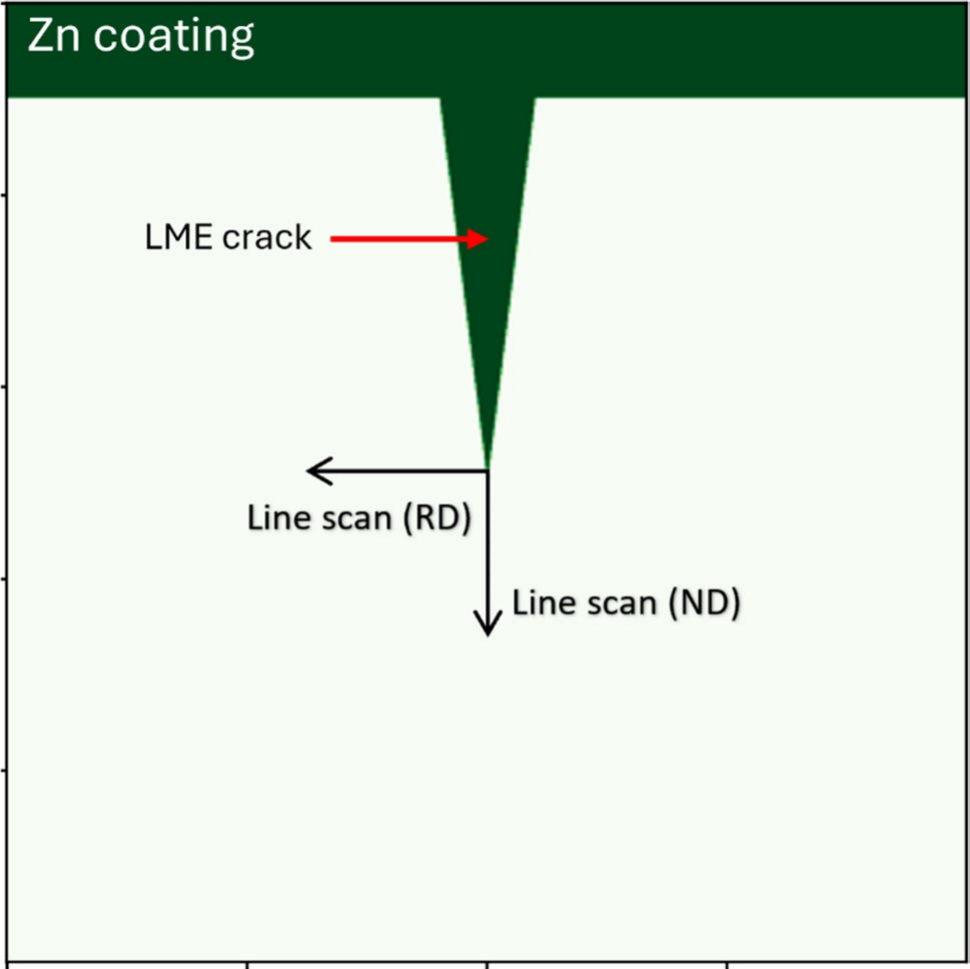
Initial condition for Zn diffusion simulation with theoretical line scans in RD and ND directions

The simulation grid consisted of 10,000 × 10,000 points, corresponding to a spatial resolution of Δ*x* = Δ*y* = 0.1 µm. For the initial condition, an LME crack is simulated as a triangular wedge filled with Zn as shown in Fig. 4. In the initial condition, the concentration of Zn in the coating and the crack is taken to be 100% and the steel to be 0%. The temporal evolution of Zn concentration was computed using time steps of Δ*t* = 1 ms with the total simulation time set to match the welding process duration. The temperature and stress values are obtained from prior experimental research by Murugan et al. [10]. According to Murugan et al., the temperature at the region of the LME cracks observed in the current study is between 650 and 1000 °C, and the maximum stress experienced in this region is 200 MPa [10]. Lower weld times (for example, 700 ms) were represented in the model by temperatures around 650–700 °C, while longer weld times (such as 1700 ms) corresponded to higher temperatures of about 850–900 °C. Although direct temperature measurements were not calculated for each weld time, the selected range represents realistic welding conditions to capture how Zn diffusion changes with increasing temperature. Since the boiling temperature of Zn is 907 °C, temperatures higher than that were not considered in the simulation, as no more liquid Zn would be available for LME.

The diffusion constant was calculated using the Arrhenius relationship in:

1 $$\begin{array}{c}D_\mathrm{eff}=D_0\mathrm{exp}\left(-\frac Q{RT}\right)\end{array}$$

where *D*_0_ is the pre-exponential diffusion factor, *Q* is the activation energy, *T* is the absolute temperature and *R* is the gas constant. The DICTRA package in ThermoCalc software was used to obtain values for *D*_0_ and *Q* for Zn in FCC-Fe.

#### Bulk diffusion modelling

The diffusion of Zn from the crack tip into the steel substrate was modelled using Fick’s second law:

2 $$\begin{array}{c}\frac{\partial C}{\partial t}=\nabla\cdot\left(D_\mathrm{eff}\nabla C\right)\end{array}$$

where *C* is the Zn concentration, *t* is time and *D* is the effective diffusion coefficient. The model assumes that the crack is fully filled with liquid Zn, which acts as an infinite source for diffusion, and that the length of the crack remains constant throughout the simulation. The formation of intermetallic phases at the Zn-steel interface is neglected.

#### Effect of stress

Stress influences diffusion by altering the chemical potential gradient, which in turn affects the flux of diffusing species [28]. The chemical potential is given by:

3 $$\begin{array}{c}\mu=\mu^0+RTlnC+\frac{\Omega\sigma}{RT}\end{array}$$

where *µ*^0^ is the reference chemical potential, *R* is the gas constant, *T* is the temperature, *σ* is the stress and *Ω* is the atomic volume. Differentiating this expression results in a modified diffusion flux equation, where the flux *J* is given as:

4 $$\begin{array}{c}J=-D\left(\nabla C+\frac\Omega{RT}C\nabla\sigma\right)\end{array}$$

Substituting this to modify Fick’s second law as:

5 $$\begin{array}{c}\frac{\partial C}{\partial t}=\nabla.\left(D\nabla C\right)+\nabla.\left(D\frac\Omega{RT}C\nabla\sigma\right)\end{array}$$

Thus, when a stress gradient is present, the effective diffusion constant *D*_eff_ can be written as:

6 $$\begin{array}{c}D_\mathrm{eff}=D_0exp\left(\frac{-Q}{RT}\right)exp\left(\frac{\Omega\nabla\sigma}{RT}\right)\end{array}$$

The contribution of the applied stress to the effective diffusion constant is $\frac{\Omega \nabla \sigma }{RT}$, which can be calculated by substituting *D*_0_ = 1.2 × 10^−5^ m^2^/s (DICTRA), *Q* = 155,522 J/mol (DICTRA), *R* = 8.314 J/mol.K, *T* = 1080 K which is the boiling temperature of Zn and the highest temperature that LME can occur, *Ω* = 1.18 × 10–29 m^3^ (ThermoCalc) and ∇*σ* = 200 MPa. It is important to clarify that Murugan et al. specifies 200MPa is the stress and not the stress gradient [10]. This stress value was conservatively used as an upper bound estimate for ∇*σ* to evaluate the possible magnitude of stress-assisted diffusion effects. This approach likely overestimates the gradient and therefore provides a conservative estimate showing that stress contributions to bulk diffusion remain negligible. The term $\frac{\Omega \nabla \sigma }{RT}$ can thus be calculated as 2.92 × 10^−25^, which is so negligible that it can be ignored completely from bulk diffusion calculations. Although stress has an important role to play in GB diffusion of Zn ahead of the crack tip [23], it has a negligible effect on the bulk diffusion which is the focus of this study and can thus be ignored in the numerical simulation of bulk Zn diffusion. Local stress concentrations near the crack tip promote atomic transport along GBs by altering chemical potential gradients [23]. Thus, even though bulk diffusion is largely unaffected by stress in the current model, stress-assisted GB diffusion is recognized as the principal mechanism driving Zn transport and crack propagation during LME.

## Results

### Characterization of LME cracks

**Fig. 5:**
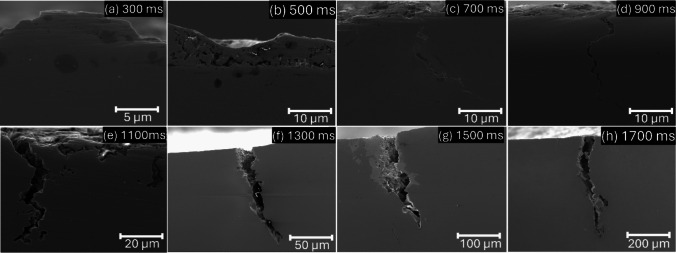
SEM micrographs of LME cracks at weld time of **a** 300 ms, **b** 500 ms, **c** 700 ms, **d** 900 ms, **e** 1100 ms, **f** 1300 ms, **g** 1500 ms and **h** 1700 ms

**Fig. 6:**
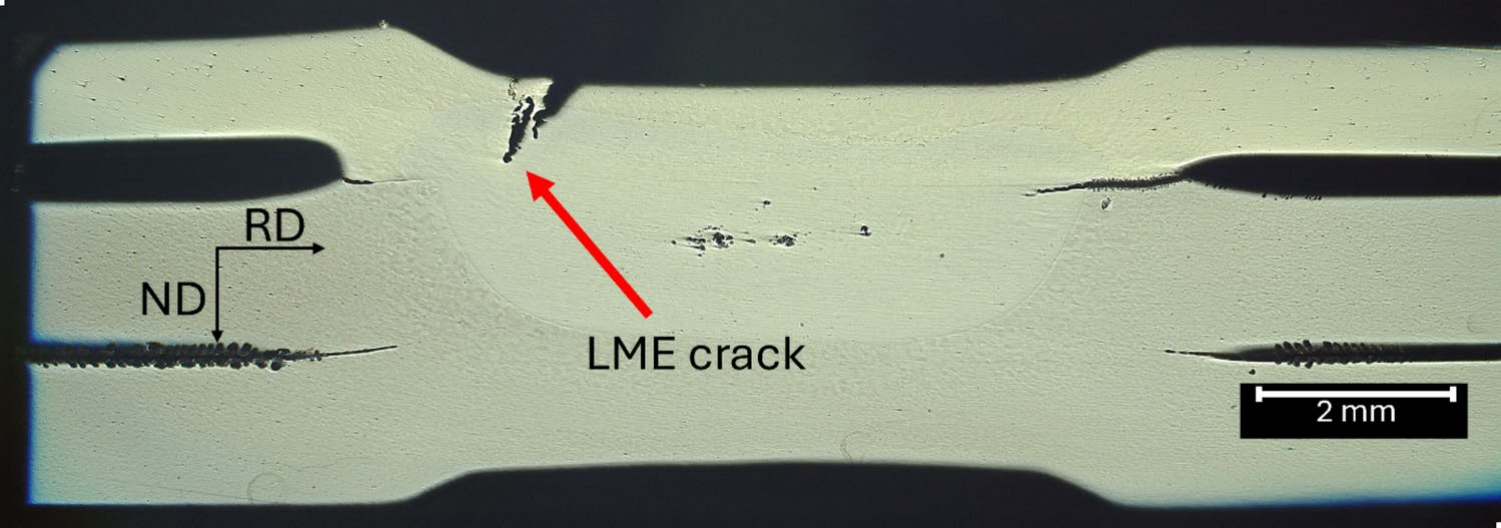
Representative optical micrograph of weld cross-section at WT = 1700 ms showing a typical LME crack in the weld shoulder region

SEM characterization was performed on all RSW samples, with the cross-section of the LME cracks at various weld times presented in Fig. 5. Most samples exhibited a single crack, with multiple cracks observed only at a WT of 1700 ms. The cracks were all identified as Type B cracks [10], which are typically associated with the weld shoulder region as shown in Fig. 6

**Fig. 7:**
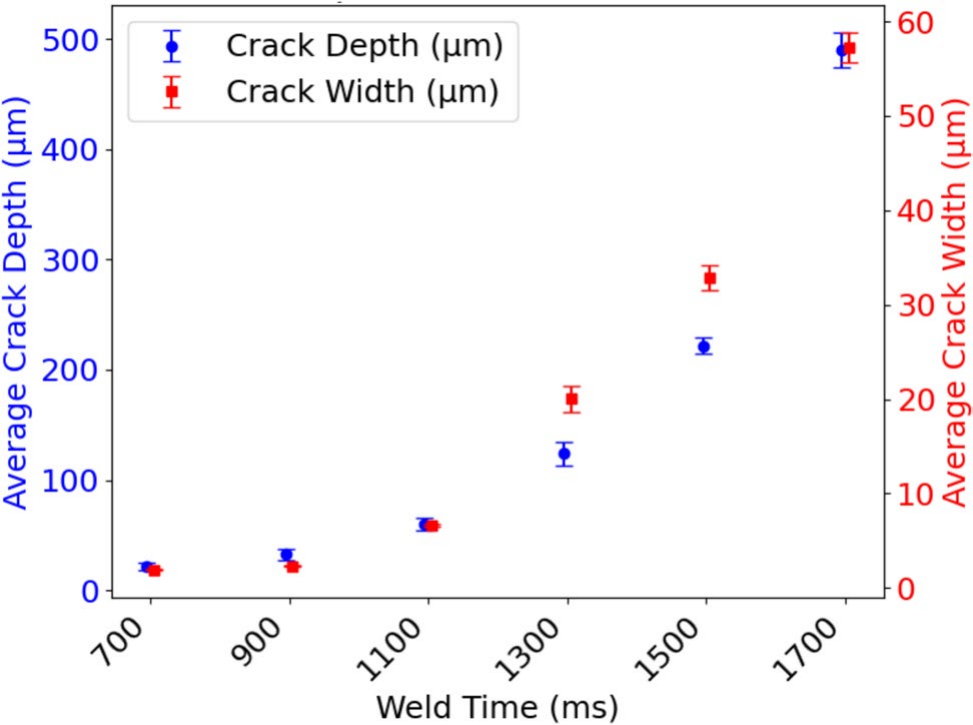
Average LME crack depth and crack width at different weld times

The average crack depth and width were found to increase with increasing weld time, as shown in Fig. 7. The crack width is measured at the opening of the crack. This trend suggests that longer weld times increase the severity of LME, possibly due to prolonged exposure to elevated temperatures and stress conditions that promote crack initiation and propagation. Cracks were not observed in samples welded for shorter durations (300 ms and 500 ms), indicating that a critical combination of temperature, stress and time is required for LME crack formation.

**Fig. 8:**
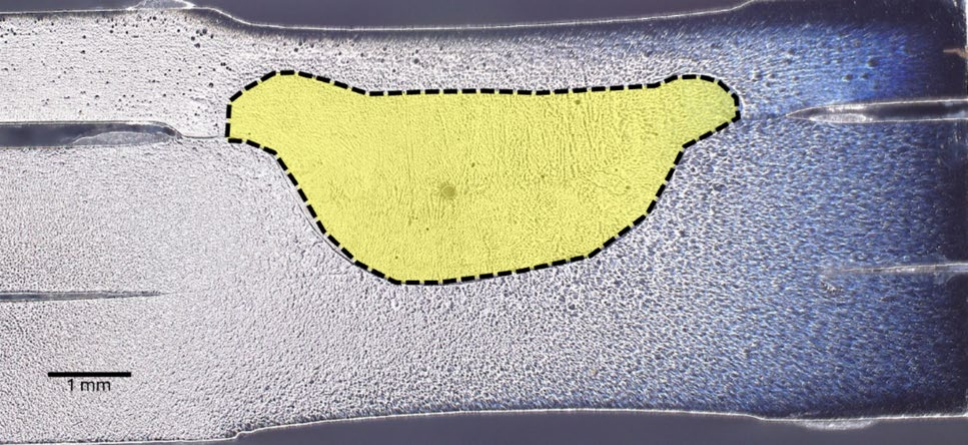
Schematic optical microscopy cross-section for weld nugget area measurement

The average weld nugget area was measured for each weld time, as shown in the schematic diagram presented in Fig. 8. The average weld nugget area is presented in Table 3. It is important to note that the nugget area could not be calculated for WT = 300 ms because the bottom DX54 sheet did not form a part of the spot weld. The heat input at this weld time was too low to sufficiently form a weld nugget at the DX54-DX54 interface. Previous studies have established that LME severity increases with increasing weld time, caused by increasing the heat input [24]. The insufficient heat input at the lower weld times of 300 and 500 ms in this study could explain the lack of observable LME.

**Table 3:** Average weld nugget area for different weld times

Weld time (ms)	Average weld nugget area (mm^2^)
300	NA
500	6.52 ± 0.23
700	9.57 ± 0.38
900	10.43 ± 0.42
1100	11.24 ± 0.65
1300	11.93 ± 0.59
1500	12.71 ± 0.82
1700	13.36 ± 0.76

### EDS analysis

**Fig. 9:**
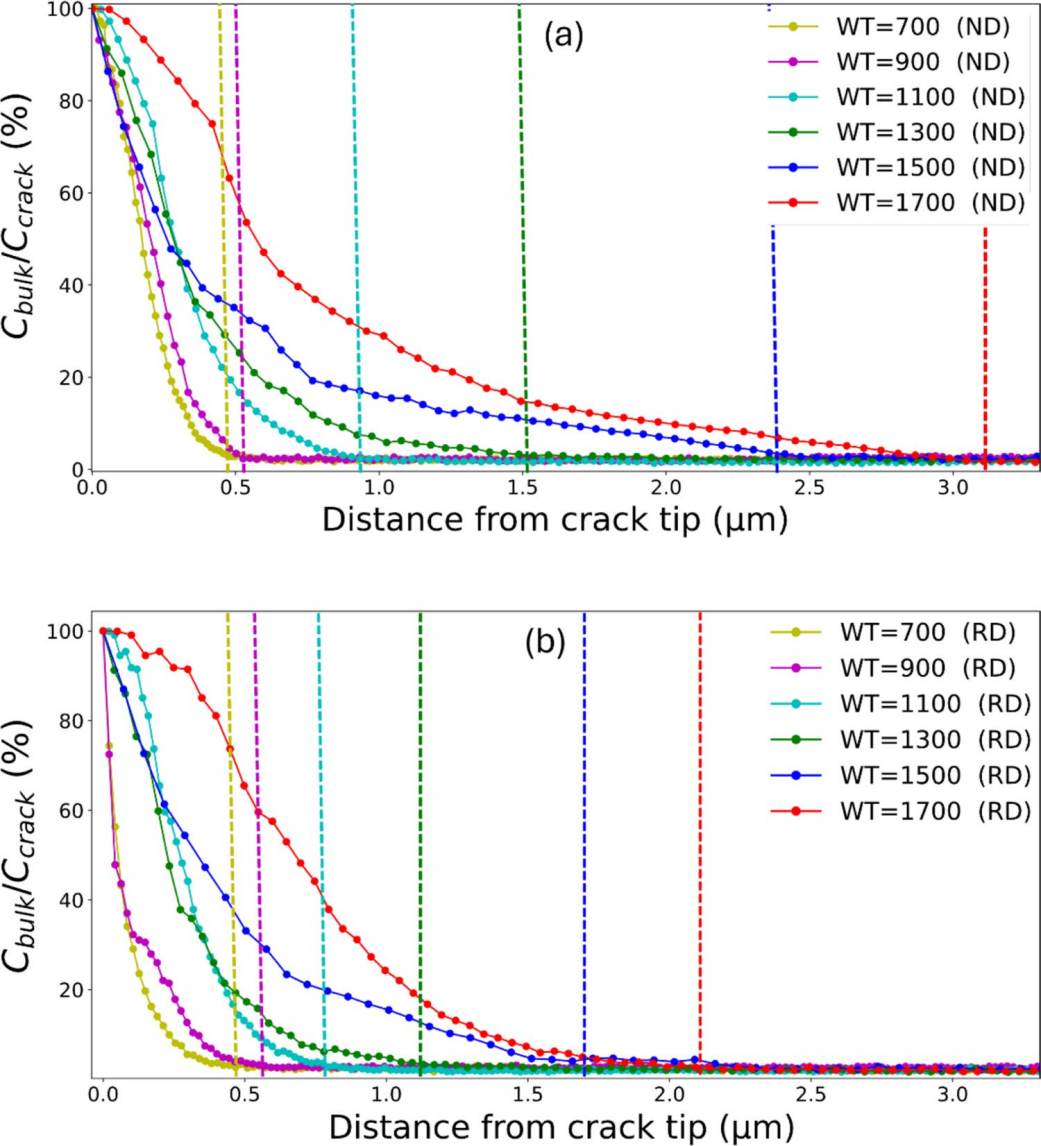
EDS Zn line scans ahead of the crack in the **a** ND direction and **b** RD direction, with the dotted lines showing Zn diffusion distance for each weld time

EDS line scans were performed for all samples with LME cracks at RSW weld times ranging from 700 to 1700 ms. Zn line scans were taken in the RD and ND directions, as described in Section 2.3 Zn diffusion profiles ahead of the crack tip in these directions were obtained, as shown in Fig. 9 for a single sample. The Zn diffusion distance, defined as the distance from the crack tip where the Zn concentration drops to zero, was calculated from these profiles. The results show a strong correlation between RSW weld time and Zn diffusion distance, with longer weld times leading to increased Zn diffusion distances in both RD and ND directions. While this result can be predicted by diffusion theory, it has not been experimentally verified in previous studies on RSW or LME.

**Fig. 10:**
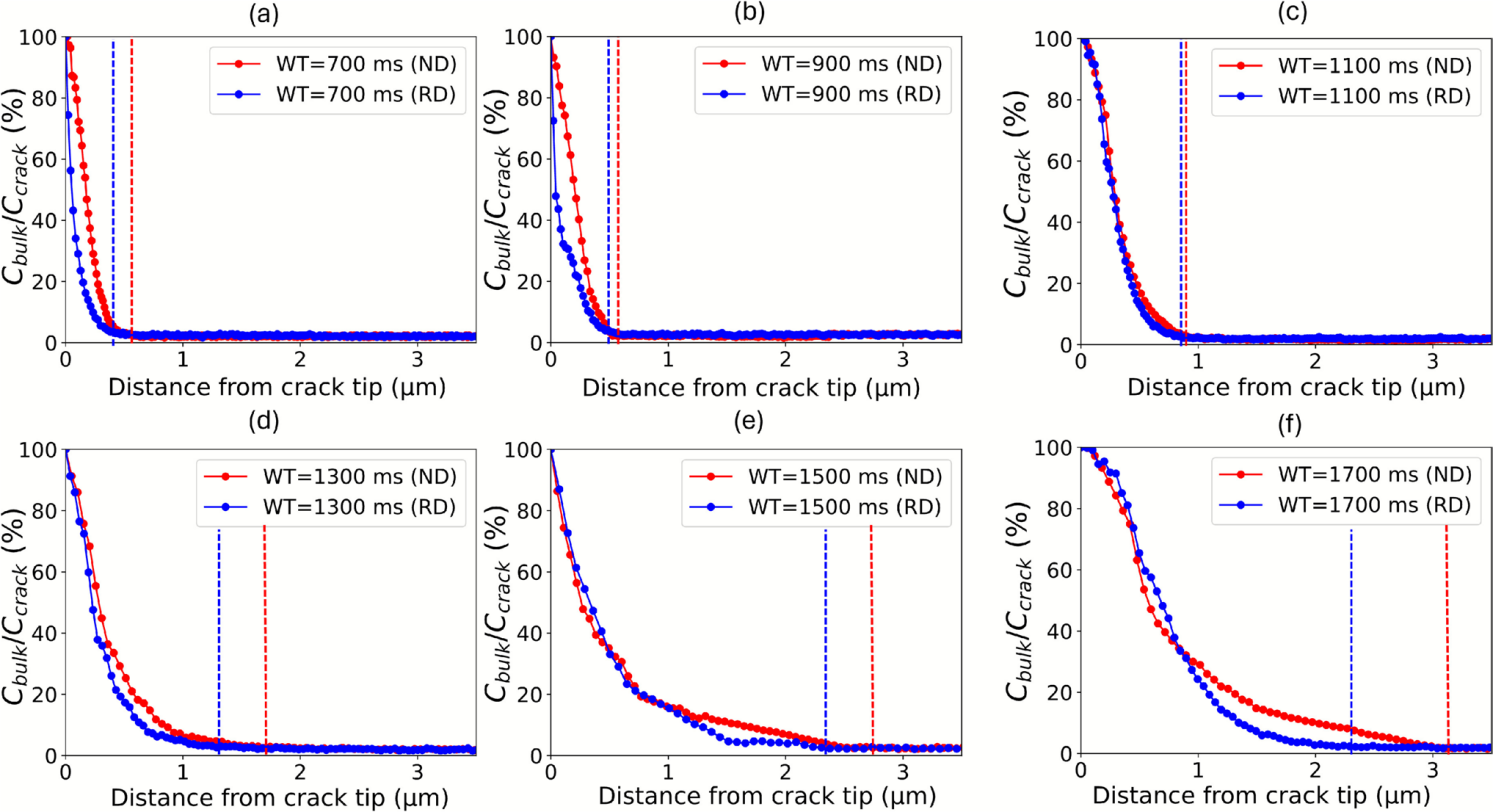
Comparing Zn diffusion profiles in ND and RD directions for one set of RSW samples at a weld time of **a** 700 ms, **b** 900 ms, **c**1100 ms, **d** 1300 ms, **e** 1500 ms and **f** 1700 ms, with the dotted lines showing Zn diffusion distance for each weld time

**Fig. 11:**
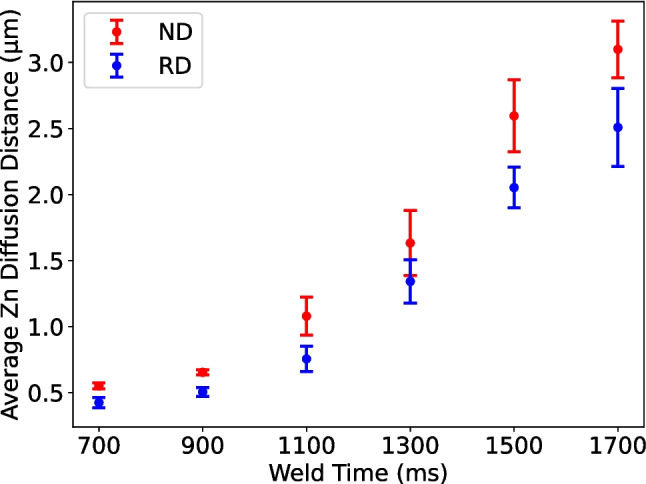
Average Zn diffusion distance measured by EDS in the ND and RD directions from the crack tip at different RSW weld times

Figure 10 compares Zn diffusion in the RD and ND directions at different weld times for a single sample, and Fig. 11 presents the average Zn diffusion distances for all four samples across different weld times. In the ND, the average Zn diffusion distance is 0.55 ± 0.01 µm at WT = 700 ms, 0.65 ± 0.01 µm at WT = 900 ms, 1.08 ± 0.07 µm at WT = 1100 ms, 1.63 ± 0.12 µm at WT = 1300 ms, 2.56 ± 0.14 µm at WT = 1500 ms and 3.09 ± 0.11 µm at WT = 1700 ms. In the RD, the average Zn diffusion distance is 0.42 ± 0.02 µm at WT = 700 ms, 0.51 ± 0.02 µm at WT = 900 ms, 0.75 ± 0.05 µm at WT = 1100 ms, 1.34 ± 0.08 µm at WT = 1300 ms, 2.05 ± 0.08 µm at WT = 1500 ms and 2.51 ± 0.14 µm at WT = 1700 ms. In all cases, the diffusion distance in the ND is greater than in the RD.

### Comparison of EDS measurements with diffusion simulations

**Fig. 12:**
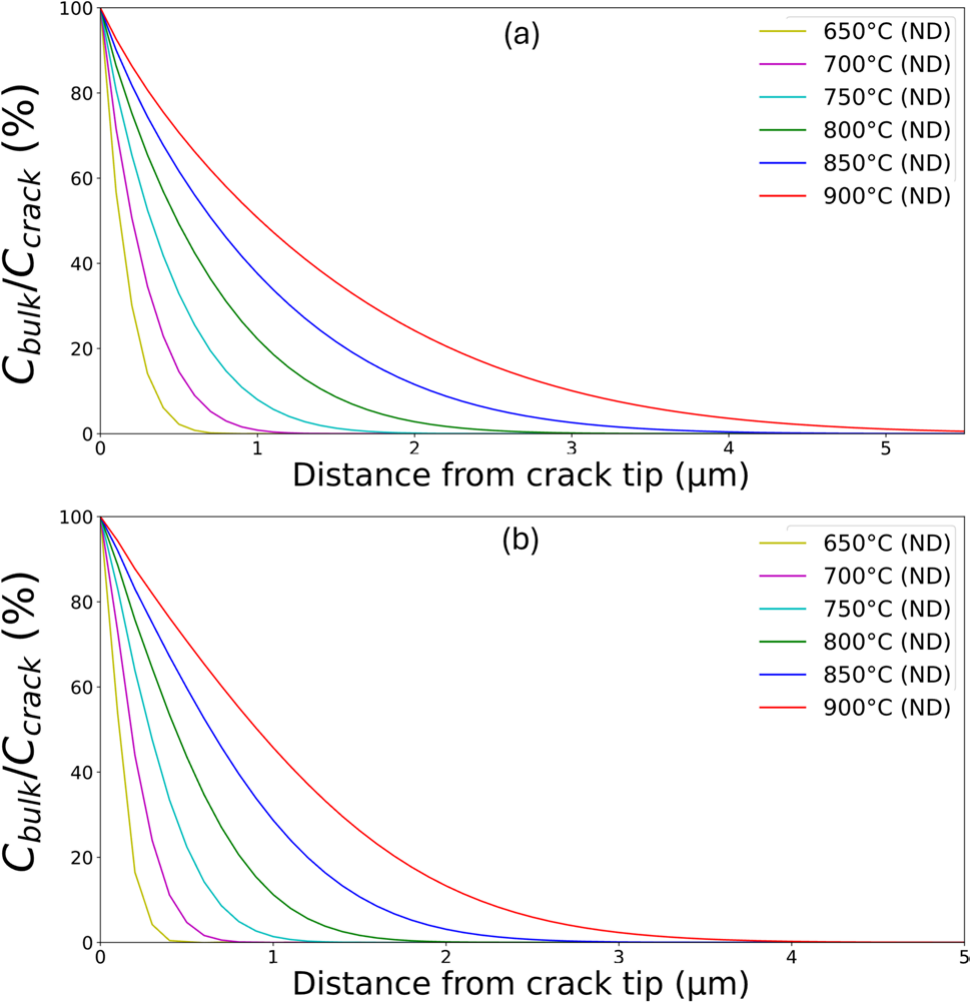
Simulated diffusion profiles of Zn from the crack tip at different temperatures in the **a** ND direction and **b** RD direction

2D diffusion modelling was performed following the methodology described in Section 2.4. These simulations generated theoretical Zn diffusion profiles in the RD and ND directions from the crack tip, analogous to the experimental EDS line scans. The diffusion profiles were modelled at temperatures of 650 °C, 700 °C, 750 °C, 800 °C, 850 °C and 900 °C, as shown in Fig. 12. While these temperatures do not directly correspond to the weld times ranging from 700 to 1700 ms, they represent a similar trend, as higher weld times are associated with increased temperatures. The objective of this comparison is to assess whether the predicted diffusion profiles from the simulations align in magnitude with the experimentally measured Zn diffusion distances.

**Fig. 13:**
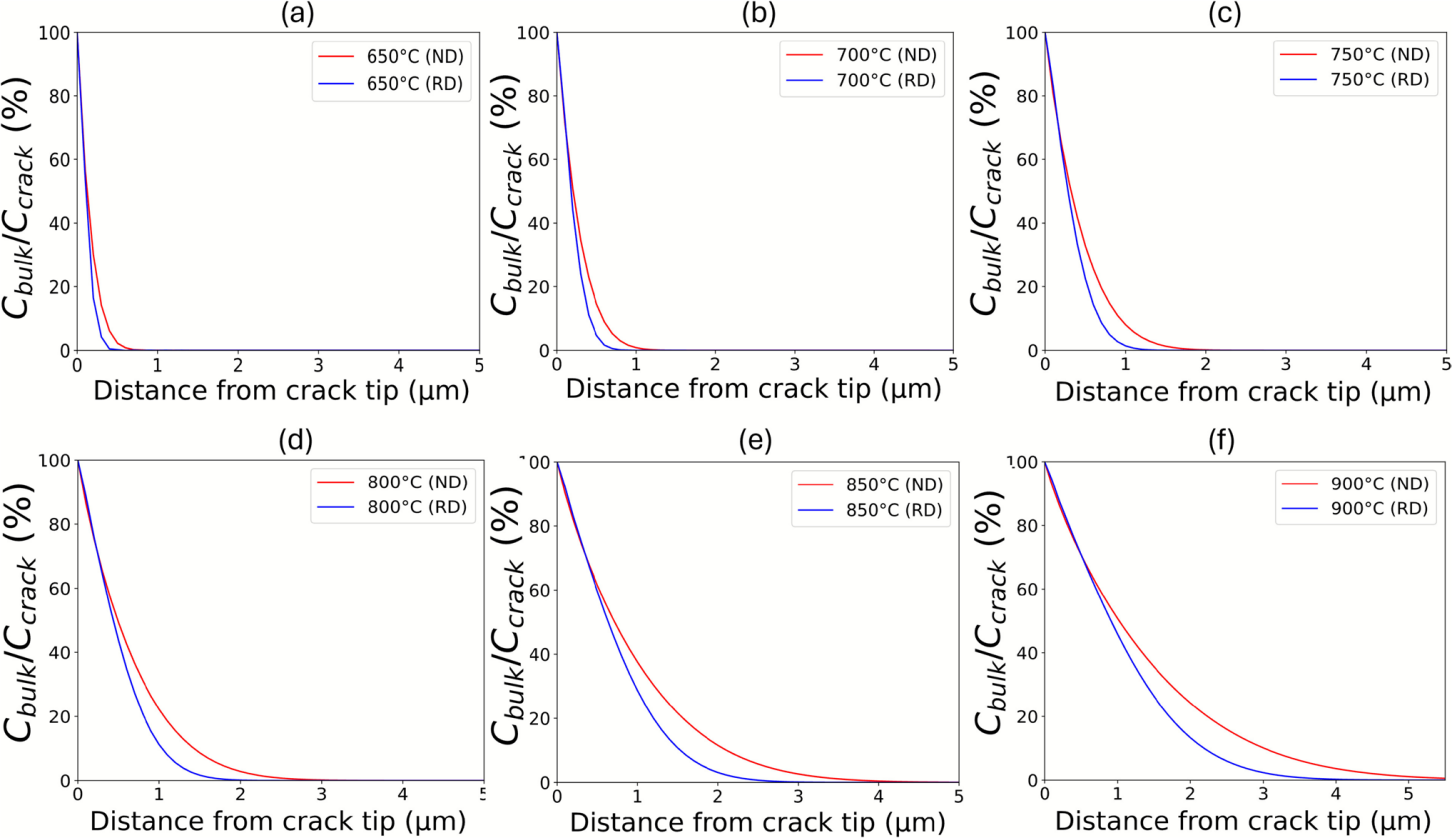
Simulated overlapping Zn diffusion profiles in the RD and ND directions from a theoretical LME crack at **a** 650 °C, **b** 700 °C, **c** 750 °C, **d** 800 °C, **e** 850 °C and **f** 900 °C

The simulation results revealed trends that are consistent with the EDS measurements by comparing the plot in Fig. 9 and Fig. 12. An increase in temperature leads to a greater Zn diffusion distance, similar to the observed relationship between weld time and Zn diffusion in the experimental results. Additionally, the simulations confirm that Zn diffusion distances in the ND direction are consistently greater than in the RD direction, as shown in Fig. 13.

The ratio of diffusion distances between the ND and RD in the simulations closely matches the experimentally observed ratios—the ratio of the average ND/RD diffusion distance for the experimentally measured EDS plots is 1.29 ± 0.09 and the same ratio for the simulated diffusion profiles is 1.42 ± 0.08. This suggests that while the model may overestimate Zn diffusion distances, it captures the relative directional differences in diffusion behaviour. A comparison of the measured and simulated Zn diffusion distances is presented in Table 4. These values were taken from the Zn diffusion distance measurements from Fig. 11 and the simulated Zn diffusion distances shown in Fig. 13.

**Table 4:** Comparison between experimentally measured and numerically simulated Zn diffusion distances

Experimentally measured results			FEA results		
Weld time (ms)	Average Zn diffusion distance RD (µm)	Average Zn diffusion distance ND (µm)	Temperature (°C)	Predicted diffusion distance RD (µm)	Predicted diffusion distance ND (µm)
700	0.42 ± 0.02	0.55 ± 0.01	650	0.4	0.6
900	0.51 ± 0.02	0.65 ± 0.01	700	0.8	1.2
1100	0.75 ± 0.05	1.08 ± 0.07	750	1.3	1.8
1300	1.34 ± 0.08	1.63 ± 0.12	800	1.9	2.7
1500	2.05 ± 0.08	2.56 ± 0.14	850	2.7	4
1700	2.51 ± 0.14	3.09 ± 0.11	900	4.1	5.2

## Discussion

### Characteristics of LME cracks in RSW

The analysis of LME crack geometry at different weld times shown in Fig. 7 indicates that crack depth increases with increasing weld time. No cracks were observed in samples welded at 300 ms and 500 ms, suggesting that a minimum threshold of temperature and stress is required for LME initiation. The weld nugget size measurements presented in Table 3 show that the nugget area increases with weld heat input, which leads to more severe LME cracking [24]. This increase in LME severity with increasing heat input has also been observed in other studies.

All observed cracks were located in the weld shoulder region which is consistent with previous studies, suggesting that the heterogeneous temperature and stress conditions in this region accelerate LME crack initiation and propagation [10, 29]. Type C cracks reported in other studies on Zn-coated TWIP steels (e.g. by Murugan et al. [10]) were not observed in the present work. This may be attributed to differences in welding conditions and joint configuration, particularly the use of a triple-stack weld with a heterogeneous material stack, which can alter local temperature gradients and stress distributions. It is possible that these cracks might be observed for the same welding conditions if more samples were analyzed.

### Comparison between EDS and diffusion modelling

Table 4 summarizes the experimentally measured Zn diffusion distances compared with the FEA predictions. These results show that both results are of the same order of magnitude—the ratio of the average ND/RD diffusion distance for the experimentally measured EDS plots is 1.29 ± 0.09 and the same ratio for the simulated diffusion profiles is 1.42 ± 0.08. The model validates directional trends and differences in diffusion between RD and ND but not the exact diffusion distances, which could be attributed to several factors. One possible source of discrepancy is the accuracy of thermodynamic data from ThermoCalc, which does not fully account for the effects of alloying elements and non-equilibrium conditions present during RSW. During RSW, the TWIP steel experiences rapid heating and cooling and non-equilibrium segregation of alloying elements such as Mn and Al. These conditions can significantly reduce the actual diffusivity of Zn compared to equilibrium predictions. As a result, the model likely overestimates diffusion distances because it assumes steady-state diffusivity values that are higher than those occurring in the spot weld. Microstructural variations such as local grain orientation and GB characteristics may introduce deviations between simulated and experimental diffusion profiles [30]. For example, GB misorientation has been observed to affect the diffusivity of alloying elements in steel. The degree of misorientation influences segregation behaviour, where certain alloying elements preferentially segregate to high-energy boundaries, locally modifying diffusion kinetics. [31]. The presence of Fe-Zn intermetallics may also affect the diffusion of Zn into the steel substrate. The formation of Fe-Zn intermetallic compounds (such as Fe_3_Zn_10_ and FeZn_10_) at the steel-Zn interface acts as a barrier to Zn transport. These intermetallic layers trap Zn atoms and limit further diffusion into the matrix [26, 30]. The simulation neglects intermetallic phase formation and treats the Zn-steel interface as a pure diffusion boundary, and does not capture the reduction in effective diffusion flux, leading to overestimated diffusion distances. The EDS results indicate that Zn diffusion distances in the ND direction are consistently greater than in the RD direction. This can be attributed to the nature of 2D diffusion and the initial conditions of the crack geometry. With a triangular crack propagating in the ND, the fundamental diffusion model predicts greater Zn penetration in this direction compared to the RD. Although the stress state differs between ND and RD, it does not influence the magnitude of bulk Zn diffusion, as demonstrated in Section 2.4.3. It is also possible that the EDS measurements capture Zn from grain boundaries in addition to bulk diffusion in the line scans. However, the spatial resolution of EDS is limited by a spot size of approximately 1 µm, while the typical GB width is on the order of a few nanometres [22]. Even if Zn segregates strongly at GBs, the signal from these nanometre-scale regions contributes less than 0.5% to the total volume sampled by the EDS beam. Therefore, the obtained line scans predominantly reflect Zn concentrations within the bulk grains rather than along GBs. This quantitative difference explains why the measured profiles correspond to bulk diffusion behaviour, while GB diffusion must be inferred indirectly [32, 33]. The directional dependence of Zn diffusion highlights the importance of crack geometry and diffusion pathways in governing Zn transport in LME-affected regions.

### Bulk and GB diffusion of Zn

Stress-assisted GB diffusion has been identified as the dominant mechanism for LME in the Fe-Zn system. According to the ‘diffusion wedge’ theory proposed by Klinger et al., Zn atoms diffuse preferentially along GBs under LME conditions while also diffusing into the bulk material. The study proposed a mathematical formulation that describes the relationship between GB and bulk diffusion, stress and the resulting concentration profiles within the diffusion wedge. The shape of this wedge is governed by the GB energy, time and the applied stress [20]. Experimental EDS analysis presented in Section 3.2 measures the Zn diffusion distance for different weld times that correspond to different temperatures and stress states. While direct measurement of GB diffusion is challenging, the analytical description of the diffusion wedge from Klinger et al. provides a way to estimate GB diffusion behaviour indirectly using the experimentally obtained bulk diffusion data. The mathematical formulation proposed by Klinger et al. was incorporated in the study by DiGiovanni et al., who modelled stress-assisted GB diffusion of Zn based on Fisher’s model of GB diffusion [23]. Their formulation describes the evolution of Zn concentration along GBs as follows:

7 $$\begin{array}{c}\frac{\partial c_\mathrm{gb}}{\partial t}=D_\mathrm{gb}\frac\partial{\partial y}\left[\frac{\partial c_\mathrm{gb}}{\partial y}-\frac{c_\mathrm{gb}\Omega}{kt}\frac{\partial\sigma}{\partial y}\right]+\frac{2D_\mathrm{bulk}}\delta\frac{\partial c}{\partial x}\end{array}$$

where *c*_gb_ is the concentration of Zn at the GB, *D*_gb_ and *D*_bulk_ are the diffusion constants at the GB and bulk, *Ω* is the atomic volume, *σ* is the stress and *δ* is the width of the GB. DiGiovanni et al. neglected the contribution of bulk diffusion based on Harrison’s classification of GB kinetics [34]. However, this reasoning is not entirely applicable to Fe-Zn LME because (a) Harrison’s classification is based on diffusion studies in alkyl halides, which do not necessarily translate to metal embrittlement mechanisms, and (b) experimental results in the present study indicate that bulk diffusion of Zn is not negligible. If the bulk diffusion term is retained in the formulation, the relationship between GB and bulk diffusion can be used to estimate GB diffusion when bulk diffusion is known.

At steady state, $\frac{\partial {c}_{\mathrm{gb}}}{\partial t}=0$, simplifying Eq. 7 to:

8 $$\begin{array}{c}D_\mathrm{gb}\frac{\partial^2c_\mathrm{gb}}{\partial y^2}+\frac{2D_\mathrm{bulk}}\delta\frac{\partial c}{\partial x}=0\end{array}$$

The GB concentration gradient can be approximated to an exponential decay according to Fisher’s model as ${c}_{\mathrm{gb}}\sim {e}^{-\frac{y}{{L}_{\mathrm{GB}}}}$ where *L*_*G*B_ is the diffusion length of Zn along the GB and can be approximated as $\frac{{\partial }^{2}{c}_{\mathrm{gb}}}{\partial {y}^{2}}\approx \frac{{c}_{\mathrm{gb}}}{{L}_{\mathrm{gb}}^{2}}$ [22]. Substituting this in the Eq. 8, the GB diffusion length can be approximated as:

9 $$\begin{array}{c}L_\mathrm{gb}\approx\left(\frac{D_\mathrm{gb}}{D_\mathrm{bulk}}\right)^\frac12L_\mathrm{bulk}\end{array}$$

where *L*_bulk_ is the measured diffusion length of Zn in the bulk. Dohie et al. experimentally measured the GB diffusion of Zn in α-Fe and developed an empirical relationship between *D*_gb_ and temperature as *D*_gb_ = −0.0034 exp(−162,000/RT) [21]. Using this, $\left(\frac{{D}_{\mathrm{gb}}}{{D}_{\mathrm{bulk}}}\right)$ is calculated to be roughly 140, based on the temperature and calculated *D*_bulk_ values from Section 2.4. However, this relationship does not account for the effect of stress on diffusion. DiGiovanni et al. developed an experimentally validated model on the effect of stress on GB diffusion, which demonstrated that under the stress gradient present in the RSW setup, the GB diffusion distance increases by a factor of 2.5 compared to the stress-free case [23]. Combining the results obtained by Dohie et al. and DiGiovanni et al. along with the calculated bulk diffusion constants, the ratio of diffusion lengths in the GB and bulk is:

10 $$\begin{array}{c}\frac{L_\mathrm{gb}}{L_\mathrm{bulk}}\approx29.58\end{array}$$

Equation 10 can thus be used to approximate GB diffusion distances at different RSW weld times from the measured EDS bulk diffusion distances—the GB diffusion distance is roughly 30 times the experimentally measured bulk diffusion distance. Thus, using bulk diffusion as an indirect measure of GB diffusion provides a reasonable approximation. A more accurate prediction can be made by integrating the effect of stress in the model predicting the GB diffusion constant of Zn in α-Fe.

This approach allows for experimental validation of theoretical LME mechanisms despite the inherent limitations of direct GB diffusion measurements. High-resolution STEM characterization by Kang et al. and Bertolo et al. provides experimental evidence demonstrating the presence of Zn in grain boundaries ahead of the crack tip [17, 35]. However, the length of the Zn diffusion region ahead of the crack tip is not fully quantified. Using bulk diffusion data to estimate GB diffusion at different temperatures and stress conditions could thus be a more effective way to quantify and understand the underlying mechanisms governing crack propagation during LME. It is important to note that although the overall diffusion profiles are comparable between EDS measurements and the numerical simulation, the study has its limitations. The resolution of EDS measurements, simulation assumptions about Zn availability and intermetallic formation and the lack of a direct correlation between weld time and temperature can be potential sources of discrepancy.

## Conclusions

This study analyzes Zn diffusion during LME in TWIP steel by experimentally measuring Zn diffusion distances in various directions over a wide range of weld times. Unlike prior LME studies where the weld time is varied, this work directly characterizes Zn diffusion profiles ahead of the crack tip and relates them to welding parameters. This methodology facilitates the investigation of the relationship between welding parameters, diffusion behaviour and the propagation of LME cracks. The key findings of this study are:

- Zn diffusion and weld time relationship: EDS measurements confirm that Zn diffusion distance increases with weld time in both RD and ND directions, with ND diffusion consistently greater than RD. This directional dependence highlights the influence of crack geometry and diffusion pathways in Zn transport ahead of the crack tip.
- Comparison between experimental and simulated diffusion: Simulated Zn diffusion distances follow the same trends as experimental data but predict higher absolute values, likely due to limitations in ThermoCalc diffusion data, intermetallic formation and other microstructural effects. However, the ratio of ND to RD diffusion distances in simulations closely matches experimental results, supporting the model’s ability to capture directional diffusion trends.
- The GB diffusion distance of Zn is approximately 30 times the bulk diffusion distance based on model approximations: Since GB diffusion is the dominant LME mechanism but difficult to quantify directly, this study demonstrates that bulk diffusion measurements can be used to estimate GB diffusion behaviour under varying weld conditions, and the GB diffusion distance of Zn is roughly 30 times the bulk diffusion distance. This represents a novel approach for indirectly quantifying GB diffusion during RSW, where direct experimental measurement remains challenging.

The diffusion trends identified in the study provide a practical foundation for process design and simulation of RSW. The validated directional dependence (ND > RD) and the estimated ratio between GB and bulk diffusion can be implemented in predictive weld models to evaluate LME risk before production trials. In this way, the study supports the development of optimized welding schedules that balance weld performance and LME resistance in Zn-coated automotive steels.

Future research should focus on directly quantifying Zn diffusion for different welding conditions along grain boundaries using high-resolution techniques such as TEM or atom probe tomography to better understand the dominant LME mechanisms. The influence of alloying elements on Zn diffusion in TWIP steel also requires further study, as existing thermodynamic databases may not fully capture non-equilibrium effects during welding. Additionally, integrating Zn diffusion models with dynamic crack propagation simulations would provide a more comprehensive understanding of LME crack growth and bridge the gap between theoretical predictions and experimental observations.

## Data Availability

The data is part of an ongoing study and can be shared upon request.

## References

[1] Candela A, Sandrini G, Gadola M, Chindamo D, Magri P (2024) Lightweighting in the automotive industry as a measure for energy efficiency: review of the main materials and methods. Heliyon 10:e29728. 10.1016/j.heliyon.2024.e29728 38681593 10.1016/j.heliyon.2024.e29728 PMC11046240

[2] Norkett JE, Dickey MD, Miller VM (2021) A review of liquid metal embrittlement: cracking open the disparate mechanisms. Metall Mater Trans A 52:2158–72. 10.1007/s11661-021-06256-y

[3] Bhattacharya D, Taylor & Francis (2018) Liquid metal embrittlement during resistance spot welding of Zn-coated high-strength steels. Mater Sci Technol 34:1809–29. 10.1080/02670836.2018.1461595

[4] Zhang H, Senkara J (2005) Resistance Welding: Fundamentals and Applications. CRC Press, Boca Raton. 10.1201/b12507

[5] Dwibedi S, Kumar B, Bhoi SR, Tripathy SR, Pattanaik S, Prasad S et al (2020) To investigate the influence of weld time on joint characteristics of Hastelloy X weldments fabricated by RSW process. Materials Today: Proceedings 26:2763–9. 10.1016/j.matpr.2020.02.576

[6] Barthelmie J, Schram A, Wesling V (2016) Liquid metal embrittlement in resistance spot welding and hot tensile tests of surface-refined TWIP steels. IOP Conf Ser Mater Sci Eng. IOP Publishing 118:012002. 10.1088/1757-899X/118/1/012002

[7] Beal C, Kleber X, Fabregue D, Bouzekri M (2012) Embrittlement of a zinc coated high manganese TWIP steel. Mater Sci Eng A 543:76–83. 10.1016/j.msea.2012.02.049

[8] Chen L, Zhao Y, Qin X (2013) Some aspects of high manganese twinning-induced plasticity (TWIP) steel, a review. Acta Metallurgica Sinica (English Letters) 26:1–15. 10.1007/s40195-012-0501-x

[9] Hong S-H, Kang J-H, Kim D, Kim S-J (2020) Si effect on Zn-assisted liquid metal embrittlement in Zn-coated TWIP steels: importance of Fe-Zn alloying reaction. Surf Coat Technol 393:125809. 10.1016/j.surfcoat.2020.125809

[10] Murugan SP, Vijayan V, Ji C, Park YD (2020) Four types of LME cracks in RSW of Zn-coated AHSS. Weld J 99:75s–92s

[11] Gordon P, An HH (1982) The mechanisms of crack initiation and crack propagation in metal-induced embrittlement of metals. Metall Trans A 13:457–472. 10.1007/BF02643354

[12] Glickman EE (2011) Dissolution condensation mechanism of stress corrosion cracking in liquid metals: driving force and crack kinetics. Metall Mater Trans A 42:250–266. 10.1007/s11661-010-0429-6

[13] Ling Z, Chen T, Kong L, Wang M, Pan H, Lei M (2019) Liquid metal embrittlement cracking during resistance spot welding of galvanized Q&P980 steel. Metall Mater Trans A 50:5128–5142. 10.1007/s11661-019-05388-6

[14] Kim YG, Kim IJ, Kim JS, Chung YI, Choi DY (2014) Evaluation of surface crack in resistance spot welds of Zn-coated steel. Mater Trans 55:171–175. 10.2320/matertrans.M2013244

[15] Ling Z, Wang M, Kong L, Chen K (2020) Towards an explanation of liquid metal embrittlement cracking in resistance spot welding of dissimilar steels. Mater Des 195:109055. 10.1016/j.matdes.2020.109055

[16] Di Giovanni C (2021) Liquid metal embrittlement in resistance spot welding: the thermomechanical origin and embrittler transport mechanism [Internet]. University of Waterloo; [cited 2022 June 29]. https://uwspace.uwaterloo.ca/handle/10012/17186. Accessed 29 June 2022

[17] Kang H, Cho L, Lee C, De Cooman BC (2016) Zn penetration in liquid metal embrittled TWIP steel. Metall Mater Trans A 47:2885–2905. 10.1007/s11661-016-3475-x

[18] Jung G, Woo IS, Suh DW, Kim S-J (2016) Liquid Zn assisted embrittlement of advanced high strength steels with different microstructures. Metals Mater Int 22:187–195. 10.1007/s12540-016-5579-7

[19] Choi D-Y, Sharma A, Uhm S-H, Jung JP (2019) Liquid metal embrittlement of resistance spot welded 1180 TRIP steel: effect of electrode force on cracking behavior. Met Mater Int 25:219–228. 10.1007/s12540-018-0180-x

[20] Klinger L, Rabkin E (2007) The effect of stress on grain boundary interdiffusion in a semi-infinite bicrystal. Acta Mater 55:4689–98. 10.1016/j.actamat.2007.04.039

[21] Dohie JS, Cahoon JR, Caley WF (2007) The grain-boundary diffusion of Zn in α-Fe. J Phase Equilib Diffus 28:322–327. 10.1007/s11669-007-9093-y

[22] Fisher JC (1951) Calculation of diffusion penetration curves for surface and grain boundary diffusion. J Appl Phys 22:74–77. 10.1063/1.1699825

[23] DiGiovanni C, Ghatei Kalashami A, Biro E, Zhou NY (2021) Liquid metal embrittlement transport mechanism in the Fe/Zn system: stress-assisted diffusion. Materialia 18:101153. 10.1016/j.mtla.2021.101153

[24] Prabitz KM, Asadzadeh MZ, Pichler M, Antretter T, Beal C, Schubert H et al (2021) Liquid metal embrittlement of advanced high strength steel: experiments and damage modeling. Materials. 10.3390/ma14185451 34576674 10.3390/ma14185451 PMC8465758

[25] Tumuluru M (2019) Effect of silicon and retained austenite on the liquid metal embrittlement cracking behavior of GEN3 and high-strength automotive steels. Weld J 98:351S-364S

[26] Ikeda Y, Yuan R, Chakraborty A, Ghassemi-Armaki H, Zuo JM, Maaß R (2022) Early stages of liquid-metal embrittlement in an advanced high-strength steel. Mater Today Adv 13:100196. 10.1016/j.mtadv.2021.100196

[27] Mehrer H, editor (2007) Solutions of the diffusion equation. Diffus Solids Fundam Methods Mater Diffus-Control Process [Internet]. Berlin, Heidelberg: Springer; [cited 2025 Feb 11]. pp 37–53. 10.1007/978-3-540-71488-0_3

[28] Shewmon P (2016) Diffusion in solids. Springer

[29] Wang X, Xie Y, Liu Z, Sun Q, Shen X, Zhang Q et al (2022) Zn-induced liquid metal embrittlement and mechanical properties of advanced high-strength steel with resistance spot weld. Mater Sci Eng A. 10.1016/j.msea.2022.143088

[30] Lee H, Jo MC, Sohn SS, Kim S-H, Song T, Kim S-K et al (2019) Microstructural evolution of liquid metal embrittlement in resistance-spot-welded galvanized TWinning-Induced Plasticity (TWIP) steel sheets. Mater Charact 147:233–41. 10.1016/j.matchar.2018.11.008

[31] Herzig C, Mishin Y (2005) Grain boundary diffusion in metals. In: Heitjans P, Kärger J, editors. Diffus Condens Matter Methods Mater Models [Internet]. Berlin, Heidelberg: Springer; [cited 2025 Oct 14]. pp 337–66. 10.1007/3-540-30970-5_8

[32] Shindo D, Oikawa T (2002) Energy dispersive X-ray spectroscopy. In: Shindo D, Oikawa T, editors. Anal Electron Microsc Mater Sci [Internet]. Tokyo: Springer Japan; [cited 2025 Oct 14]. pp 81–102. 10.1007/978-4-431-66988-3_4

[33] Lejček P (2010) Grain boundaries: description, structure and thermodynamics. In: Lejcek P, editor. Grain Bound Segreg Met [Internet]. Berlin, Heidelberg: Springer; [cited 2025 Oct 14]. pp 5–24. 10.1007/978-3-642-12505-8_2

[34] Harrison LG (1961) Influence of dislocations on diffusion kinetics in solids with particular reference to the alkali halides. Trans Faraday Soc 57:1191–1199. 10.1039/TF9615701191

[35] Bertolo V, Mahadevan G, de Kloe R, Petrov RH, Popovich V (2025) Decoupling early-stage cracking and propagation mechanisms in liquid metal embrittlement of Zn-galvanised TWIP steel. J Mater Res Technol 38:1617–32. 10.1016/j.jmrt.2025.08.055

